# Automated Retrieval of Heterogeneous Proteomic Data for Machine Learning

**DOI:** 10.3390/jpm13050790

**Published:** 2023-05-02

**Authors:** Abdul Rafay, Muzzamil Aziz, Amjad Zia, Abdul R. Asif

**Affiliations:** 1 Department for Clinical Chemistry/Interdisciplinary UMG Laboratories, University Medical Center, 37075 Göttingen, Germany; 2 Future Networks, eScience Group, Gesellschaft für Wissenschaftliche Datenverarbeitung mbH Göttingen (GWDG), 37077 Göttingen, Germany; 3 German Centre for Cardiovascular Research (DZHK), Partner Site Göttingen, 37075 Göttingen, Germany

**Keywords:** mass spectrometry, proteomics, machine learning, data scraping, data harvesting

## Abstract

Proteomics instrumentation and the corresponding bioinformatics tools have evolved at a rapid pace in the last 20 years, whereas the exploitation of deep learning techniques in proteomics is on the horizon. The ability to revisit proteomics raw data, in particular, could be a valuable resource for machine learning applications seeking new insight into protein expression and functions of previously acquired data from different instruments under various lab conditions. We map publicly available proteomics repositories (such as ProteomeXchange) and relevant publications to extract MS/MS data to form one large database that contains the patient history and mass spectrometric data acquired for the patient sample. The extracted mapped dataset should enable the research to overcome the issues attached to the dispersions of proteomics data on the internet, which makes it difficult to apply emerging new bioinformatics tools and deep learning algorithms. The workflow proposed in this study enables a linked large dataset of heart-related proteomics data, which could be easily and efficiently applied to machine learning and deep learning algorithms for futuristic predictions of heart diseases and modeling. Data scraping and crawling offer a powerful tool to harvest and prepare the training and test datasets; however, the authors advocate caution because of ethical and legal issues, as well as the need to ensure the quality and accuracy of the data that are being collected.

## 1. Introduction

The revolution in mass spectrometric technologies since the early 1990s allowed the identification and quantification of a larger number of proteins in a variety of sample types. In the last 10 years, there have been significant advancements in high-resolution mass spectrometry (HRMS), which took the identification and quantification of proteins to a higher level of accuracy and sensitivity [1]. However, the accumulation of MS data possesses numerous challenges including vender-specific data formats and data heterogeneity with regard to the quality and completeness of the data generated by different MS instruments (Table 1). For instance, there might be events where the data generated by some of the instruments is of low quality and with a high level of background noise, while others may produce high-quality data with a low level of background noise. In addition, some instruments may have different levels of sensitivity and specificity for proteomics analysis, which ultimately impacts the accuracy and completeness of the data produced from these instruments and from different labs [2].

Machine learning (ML) and deep learning (DL) models perform significantly better when trained on large datasets to identify more complex patterns and relationships within the data. The rapid growth in protein sequencing technology has led to enormous amounts of proteome data, which present a valuable opportunity for machine learning applications in the field of bioinformatics. Proteome data provide information on protein expression, modification, and function, and can be used to predict the effects of genetic mutations and drug treatments. However, the sheer volume of proteome data can pose a significant challenge for analysis and interpretation. In the last two decades, the field of deep learning has made remarkable strides, outperforming conventional machine learning algorithms in a variety of fields, including computer vision, speech recognition, natural language processing, bioinformatics, and medical image analysis. In an effort to mimic the functioning of the human brain, deep learning makes use of the capabilities of artificial neural networks that assist representation learning. Deep learning can automatically discover and learn complex patterns and features from data, unlike traditional machine learning approaches such as support vector machines (SVM) and random forests (RF), which require manual feature engineering. As a result, it works especially well for scientific fields that have access to huge, complex datasets.

With the advancement of MS instruments, proteomics data are increasing at a rapid pace. One source of proteome data that has gained significant attention in recent years is the Protein Data Bank (PDB), which contains over 175,326 entries only for protein structures (access date: 28 February 2023). Similarly, the current release of UniProtKB/Swiss-Prot protein knowledgebase statistics (release 2023_01) showed a huge amount of additional data from the previous year (2022). These sources provide a rich foundation of proteomics data for machine learning algorithms, but the diversity of the data, as well as their size, poses significant challenges for processing and analysis [3]. Therefore, it is crucial to integrate the pile of scattered proteomics data into a single database—a rapidly developing field called “proteomic data integration”—for improving our comprehension of complex biological systems and for creating new diagnostics and therapeutics approaches. 

In liquid-chromatography mass spectrometry (LC-MS)-based proteomics, discovery experiments were often employed to investigate potential biomarker candidates. While fold change and statistical significance are still regularly utilized, supervised machine learning (ML) is increasingly becoming acknowledged as a potent method for identifying potential biomarker candidates. ML methods like logistic regression (LR), random forest (RF), K-nearest neighbors (KNN), and support vector machine (SVM) are being used by proteomics researchers. These ML techniques have been successfully used to identify biomarkers for the development of cancer and forecast its subtypes. These ML techniques can be used in a variety of platforms, including Perseus, the R, or Python programming languages and commercially available applications [4].

To address the above-mentioned challenges, AI tools and technologies are being developed to enable the efficient processing and analysis of proteome data. For instance, deep learning techniques such as convolutional neural networks and recurrent neural networks have shown promise in analyzing large-scale proteome data [5]. Additionally, cloud-based technologies such as Apache Spark and Hadoop can be used to parallelize data processing and facilitate data sharing among different researchers [6]. 

The use of AI tools and technologies for the analysis of proteome data holds significant promise for unlocking the full potential of these valuable resources. However, it is important to ensure that the ethical and legal aspects are considered and that the quality and accuracy of the data are rigorously evaluated. By leveraging the power of AI and machine learning, researchers can make significant strides in understanding the complexities of proteomics data and using them to develop new treatments and therapies. In the present study, we collect the raw MS data from 126 published studies using heart tissue/cell material proteomics from the ProteomeXchange repository. The data were allied with the patient/animal disease and treatment history. For MS data submitted without history information, the corresponding authors were automatically sent email reminders to update the missing information. 

## 2. Methodology

**Figure 1:**
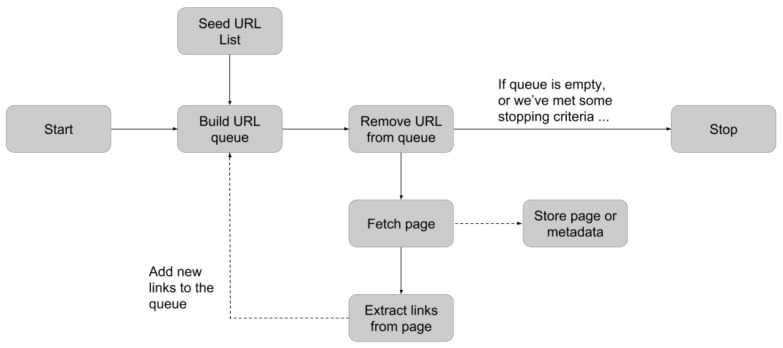
An illustration of data crawling. In the automatic crawling and scraping, the web bot takes the provided keywords, and then, it crawls all the links fetched from the main web page and builds a queue. Each URL is deque from the queue and the web scraper works on that URL.

The Python programming language was used to automate the web crawling and scraping processes for the ProteomeXchange website and publication web pages. To help implement the web crawler spider and parse HTML data, the Scrapy framework (v. 2.8.0) of Python (v. 3.10) was employed. The main page of the ProteomeXchange website contains an interactive form to input the search criteria and narrows down the publications matching the search criteria. The web source (ProteomeXchange) contained thousands of proteome-related studies, and only those studies related to heart diseases were targeted. The Selenium web driver (v. 4.8.3) and Python framework were used to create a web bot that can automatically fill in the form to make a search engine work. The web crawling layout utilized in this project is depicted in Figure 1. A web bot is used to take the provided keywords and crawl through the links that are downloadable from the main page during the automatic crawling and scraping process. Each URL is then added to a queue as a result of this action. The URLs are then taken out of the queue one at a time, and the web scraper starts working on each URL individually to extract the necessary data. This procedure makes it possible to efficiently and methodically extract data from a large number of web pages, allowing for the timely and efficient gathering of enormous amounts of information.

### 2.1. Data Request 

With the keyword “heart”, a total of 280 publications were scraped (access date: 23 January 2023), and only eight datasets (publications) included patient medical histories. Thus, an automated email remainder was created and sent to the corresponding authors of the corresponding publications, requesting data on a patient’s medical history and experimental design. The whole pipeline was automated using Python programming and consolidated to fetch the author, corresponding author’s email, and publication details. The libraries, packages, and their versions that were used are as follows: Scrapy (V. 2.8.0), Selenium web driver (v. 4.8.3), Pandas (v. 1.5.3), Python (v. 3.10), and Dask (v. 2023.3.2).

### 2.2. Data Collection 

Python, along with its powerful libraries such as Pandas and NumPy, is a popular choice for processing, analyzing, and visualizing large datasets. The Pandas library provides a comprehensive set of data structures and functions for working with structured data, while the NumPy library offers fast numerical operations on large arrays. To scrape data from the website, Scrapy with Python was used, which is considered a fast and powerful Python-based web crawling framework in comparison to other tools [7]. 

**Figure 2:**
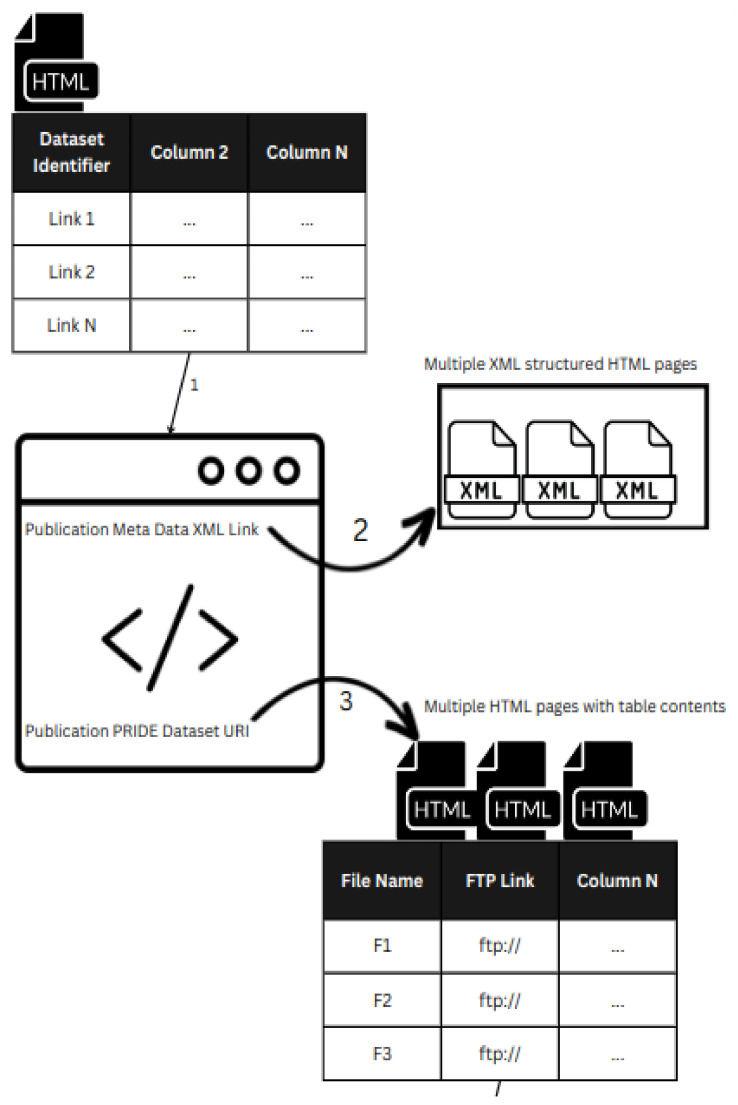
An illustration of the data collection. First, we have a master table scraped from the main web page based on the given keyword filter. Each URI present in the master table is used to extract the publication information from the online XML page as shown in step 1. In step 2, the scraper iterates through the nested XML documents and multiple HTML tables present on the web page. At the end, it extracts all the necessary information and stores it in the master table.

We first iterated through the table content present on the first page and harvested the web links to their personalized publication metadata web page. The personalized publication metadata web page contained the publication-related information in XML format, which was also scraped, and also included the publication link and the dataset PRIDE URI, as well as an FTP link to the RAW proteome spectra files. Furthermore, the profiled page also contained the FTP link to the experimental design file, which was present in the TSV file format. All the heterogeneous data were scraped from multiple sources and combined into one big CSV file in a wide columnar way, which contains a number of columns with respective data in them. The scraping steps are demonstrated in Figure 2. 

The process of data collection can be illustrated in two steps (Figure 2). First, a master table is made on the primary web page using web scraping techniques based on the specified keyword filter. The publication data is then extracted from the online extensible markup language (XML) page using each uniform resource identifier (URI) present in the master table, as shown in step 1. The web scraper iterates through the numerous hypertext markup language (HTML) tables and nested XML documents that are present on the web page in the second step. The master table contains all the data that the scraper gathers from the HTML tables. By following these steps, the necessary data are gathered and stored in an organized manner, making them ready for additional analysis and processing. 

### 2.3. Data Preparation and Data Wrangling 

The harvested data were checked for errors, and data wrangling and munging procedures were applied, including deleting duplicate entries, resolving missing or inconsistent values, translating the data into a standard format, and dealing with outliers or mistakes, to ensure the data are organized, consistent, and acceptable for the analysis. The data crawled and scraped from web pages were unstructured and in raw format. Python Numpy, Pandas (v. 1.5.3), and Dask libraries (v. 2023.3.2) were applied for the wrangling and munging of the data. Some fields based on the mathematical formulas were also derived to obtain the correct and meaningful attributes to make a machine learning model on it, e.g., age column was derived from date-of-birth column. There was also a great noise in the data, such as many attributes coming with different column names and containing uncertain values, which were also managed through Python and cleaned with Dask and Pandas.

### 2.4. Data Consolidation 

To store, manage, and analyze the data as a single entity, it is necessary to consolidate the data from several sources into a single database. We adopted data lake architecture and summarized the main data sources to be three when attributed, and they are as follows:

- Publication metadata: it refers to the information related to the publication itself. For example, authors’ names and emails, titles, instruments used, lab heads, keywords, etc.
- Publication characteristics and relevant information: it refers to the published dataset of the publication and information related to it. For example, the FTP file link and file types and formats of users observed measurements of proteome spectra available in RAW file formats.
- Medical patient history: it refers to the information related to the observed patients. For example, patient age, gender, cell types, observed values, protein sequences, disease type, etc.

**Figure 3:**
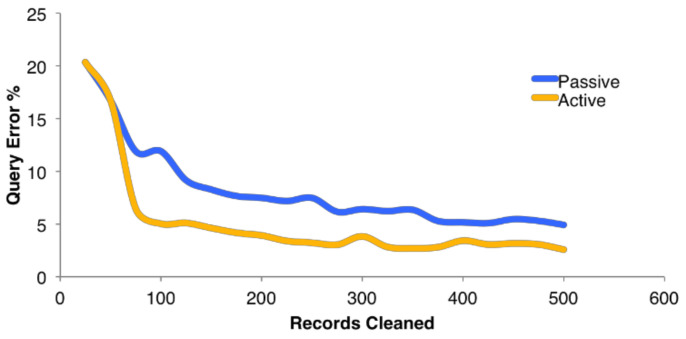
Illustration of average records cleaned per publication. The X-axis represents the number of records cleaned in contrast to percentage of query error on *Y*-axis.

Figure 3 depicts the data collection and cleaning pipeline statistics. The X-axis represents the number of records cleaned per publication, while the Y-axis represents the percentage of query errors. The graph is divided into two sections: passive and active cleaning. Passive cleaning is the process of removing unnecessary information or duplicates from a dataset without requiring user intervention, whereas active cleaning requires user input to identify and remove irrelevant data. The graph demonstrates that for both passive and active cleaning, the percentage of query errors tends to decline as the number of records cleaned rises. This suggests that thorough cleaning produces data of higher quality that can be amassed. The graph also demonstrates that active cleaning, as opposed to passive cleaning, tends to produce a lower percentage of query errors. This further suggests that adding user input can enhance the precision and standard of the data-cleaning process.

### 2.5. Proposed Architecture for Machine Learning Exploitation 

**Figure 4:**
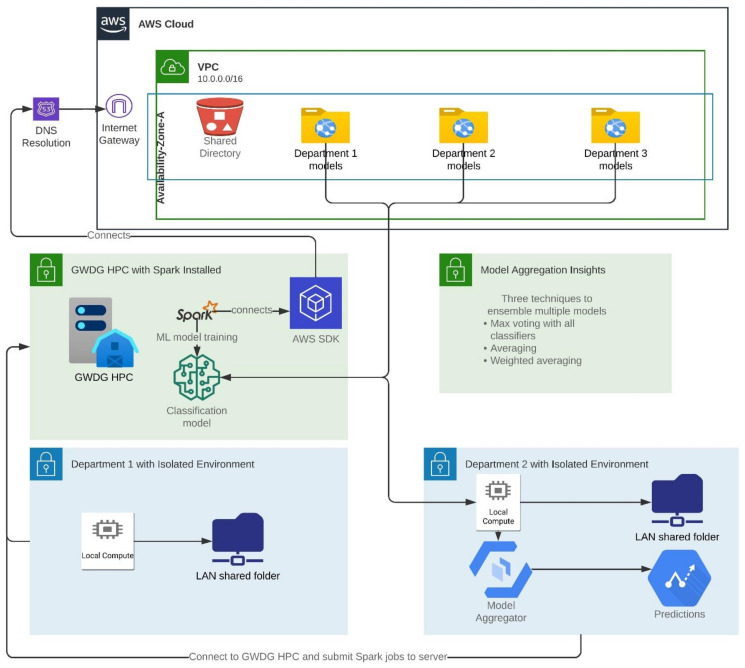
Architectural design of the project. The architecture represents the logical design of the project. Each bucket on the AWS S3 buckets service depicts a department-trained model, stores new trained models, and makes time travel to each model easier. Every department has shared access to the GWDG HPC system, where spark machine learning conducted on datasets and trained models is pushed to AWS S3 buckets. This serves the purpose of federated machine learning. All the buckets with trained models are used to make one voting classifier model to have more robust results.

Here, we propose exemplary architecture for machine learning and deep learning models to exploit the consolidated cleaned data (Figure 4). In this proposed architecture, AWS S3 buckets can be employed to store data and form a data lake on the cloud for fast writing and data retrieval. The collected data are already more than one terabyte and with several heart diseases; therefore, Apache Spark (v. 3.1) can be adopted to train the model in a cluster by utilizing the Spark Gang Scheduler. Our fetched data are from multiple publications; therefore, the data of each publication will be treated as one department to train a separate model on the data and then push that trained model to the S3 object store. In a similar way, a separate model of each publication will be available in the respective folder on the cloud. Later on, all the trained models for a disease will be consolidated and merged into one robust model as a federated machine learning model. 

The architectural design of the project illustrated in Figure 4 is the logical layout of the system. AWS S3 buckets are used to store the department-trained models, which makes it easier to keep track of the time travel of each model. Each department has shared access to the GWDG high-performance computing (HPC) system, where Spark ML is performed on the datasets, and the trained models are then pushed to the AWS S3 buckets. This approach enables federated machine learning, which is a distributed approach to machine learning. All the buckets containing the trained models are then used to create a voting classifier model, which produces more robust results. This design allows for the efficient storage, sharing, and processing of data and models across departments, facilitating collaboration and improving the overall performance of the system. 

### 2.6. Challenges

The challenges faced during scraping and crawling publicly available resources are as follows:

- *Website structure:* different websites and web pages have different structures, which make it challenging to extract data in a consistent and reliable manner. This results in the need to write separate scripts for each website and for its nested web pages.
- *Dynamic content:* some web pages use dynamic content, such as JavaScript, to load or update their pages. This makes it difficult to scrape the content in its final form, as the data are generated after the page has loaded. We tackle this problem of dynamic content through a Selenium bot.
- *Data quality:* data obtained through scraping were incomplete and contained errors, making it necessary to validate and clean the data before they could be used.
- *Incompleteness:* data were incomplete, which leads to missing values, inconsistent data, and a lack of information for certain fields. This made it difficult to clean and prepare the data for analysis. We resolved the challenge with the help of the rich functions available for data manipulation in Dask and Pandas.
- *Duplicates:* data contained duplicates, which lead to errors in analysis and cause issues when trying to merge data from multiple sources. We leveraged the rich functions in Pandas and Dask to handle the duplicates.
- *Incorrect data types:* data can be stored in the wrong format, such as a string when it should be a date, or a number when it should be a categorical variable. This causes issues when trying to perform data operations. We convert the data types with the help of the Dask Dataframe functions.

## 3. Discussion

We scraped the data of 280 publications related to heart diseases from ProteomeXchange and all its nested databases/webpages. The data come from multiple heterogeneous sources and with a lot of noise. We use Python programming language to crawl and scrape data and make use of Numpy, Pandas, and Dask frameworks to clean the data and formulate them in a format that is ready to prepare a machine learning model. The pipelines totally replace the huge manual effort needed by a human to make consolidated data. The pipeline’s running time is in minutes to scrape any number of publications, as it runs in a multi-threaded environment. The importance and benefits of this large and single repository for proteome data extend to the data that have been scraped from many websites and research studies are increased data accessibility, improved data quality, enhanced data analysis, reduced redundancy, and facilitated collaboration. By collecting and compiling data from a multitude of sources, a comprehensive and diverse dataset is created that can be utilized by researchers to train and develop machine learning models. The use of machine learning models in proteomics has the potential to revolutionize the field by providing more accurate and precise predictions and classifications of proteome data. The use of such models can also lead to the discovery of novel biomarkers and therapeutic targets for a wide range of diseases. Furthermore, the creation of a single repository for proteome data scraped from various sources promotes collaboration and the sharing of resources among researchers, which can lead to breakthroughs and advancements that may not have been possible otherwise. Overall, a large and single repository for proteome data scraped from many websites and research studies is an essential resource for researchers who aim to utilize AI and machine learning in proteomics research.

To the best of our knowledge, this is the first time that a single source or file of proteomics data has been compiled specifically for research on heart diseases. By scraping data from a wide range of sources and consolidating them into a single repository, we have created a unique and valuable resource for researchers in this field. This comprehensive dataset provides a significant advantage for researchers who previously had to manually gather and curate proteomics data from multiple sources. With this new resource, researchers can quickly and easily access a diverse range of proteomics data related to heart diseases, which can lead to new insights, discoveries, and advancements in the field.

Several proteomics data repositories and multi-omics integration platforms are publicly available for researchers with various purposes and functionalities, e.g., ProteomXchange [8], the Mass Spectrometry Interactive Virtual Environment (MassIVE, https://massive.ucsd.edu/ProteoSAFe/static/massive.jsp accessed on 26 April 2023), jPOST [9], PRoteomics IDEntifications (PRIDE) [10], PeptideAtlas SRM Experiment Library (PeptideAtlas/PASSEL) [11], Panorama Public [12], and iProX [13]. The PRIDE database is available with the aim to archive various types of proteomics mass spectrometry data for reproducible research, facilitate protein-centric integration of MS-based data for variant and modification analysis, and furnish MS-based expression data to the Expression Atlas [10]. An integrated proteomics data system (IPDS), a data integration platform, is developed to collect the expanding heterogeneous proteomic data and its relevant information and to make this information easily accessible to the scientific community [6]. Despite all these and other databases, to our best knowledge, there is no publicly available dataset/repository dedicated to a biomedical data integration system that is curated especially for a machine learning point of view, where all MS files are mapped to its respective patient medical history without personal information disclosure.

Accessing multiple data sources has long been a study area in the realm of multi-database and data integration systems. With the advent of cloud databases and large data processing frameworks, however, the solution has expanded to multistore systems. By utilizing a shared query engine and, in some circumstances, a data processing framework like Spark, these systems provide consolidated access to multiple data stores, such as the Relational Database Management System (RDBMS), NoSQL, and Hadoop Distributed File System (HDFS). In a nutshell, multistore systems enable seamless access to many types of data sources through the use of a common querying and processing approach, making it easier to analyze and extract insights from data stored in different forms and locations [6]. 

Big data technologies-based integration systems are created to handle and combine massive, complicated datasets from various sources, facilitating effective querying and analysis of various data sources. These systems are vital for large data processing and offer researchers effective tools for delving into intricate biological processes. Several types of big data technology-based integration systems are available, such as query-optimization-based systems (QoX and Estocada), distributed-processing-based systems (BigIntegrator and MyriaDB), hybrid systems (HadoopDB), and schema-free query engines (Apache Drill). Our system is using Apache Spark instead of Hadoop or SQL. This is because the emergence of big data frameworks has led to the development of several SQL-like query processing systems, including Apache Spark. An open-source engine called Spark is particularly adept at handling and analyzing massive amounts of data, and it can access information from many different sources, including HDFS, OpenStack Swift, Amazon S3, and Cassandra. Both in memory and disk operations, Spark performs noticeably better than Hadoop MapReduce [14,15].

In summary, the collection and analysis of proteome data using machine learning has the potential to improve the accuracy of diagnosis and prediction of heart diseases. These studies demonstrate the potential of already acquired proteome data for the machine learning purpose to improve our understanding of diseases without need to consume further precious patient samples to improve the treatment and prevention strategies. 

## Figures and Tables

**Table 1:** Various MS output file formats encoding the mass spectra.

Files Formats		
Vendor Specific		Open Spectrum
d/	(Waters and Agilent instruments)	mzXML
raw/	(Waters and Agilent instruments)	mzML
RAW	(Thermo Scientific instruments)	mz5
yep	(Agilent/Bruker instruments)	imzML
wiff	(AB SCIEX instruments)	mzData
t2d	(ABI/Sciex instruments)	YAMSF
		MGF
		dta
		pkl
		ms2
		ms1

## Data Availability

https://github.com/FutureSceince/Automated-Retrieval-of-Heterogeneous-Proteomic-Data-for-Machine-Learning (accessed on 26 April 2023).
